# Surface modifications of carbon nanodots reveal the chemical source of their bright fluorescence†

**DOI:** 10.1039/d0na00871k

**Published:** 2020-12-10

**Authors:** Asmita Dutta, Shimon T. Y. Trolles-Cavalcante, Annie Cleetus, Vered Marks, Alex Schechter, Richard D. Webster, Arie Borenstein

**Affiliations:** Department of Chemical Sciences, Ariel University Ariel Israel arieb@ariel.ac.il; Division of Chemistry & Biological Chemistry, School of Physical and Mathematical Sciences, Nanyang Technological University 637371 Singapore

## Abstract

Fluorescent carbon nanodots (CNDs) have drawn increasing attention in recent years. These cost-effective and eco-friendly nanomaterials with bright fluorescence have been investigated as promising materials for electrooptic and bioimaging applications. However, the chemical source stimulating their strong fluorescence has not been completely identified to date. Depending on the chemical composition, two absorption peaks are observed in the visible range. In this study, we applied selected chemical modifications to CNDs in order to elucidate the correlation between the chemical structure and optical behavior of CNDs. Varying the amount of acetic acid in the synthesis process resulted in different effects on the absorbance and fluorescence photo-spectra. Specifically, at a low concentration (10%), the fluorescence is dramatically red shifted from 340 to 405 nm. Comprehensive characterization of the chemical modification by FTIR and XPS allows identification of the role of acetic acid in the reaction mechanism leading to the modified photoactivity. The functional group responsible for the 405 nm peak was identified as HPPT. We describe a chemical mechanism involving acetic acid that leads to an increased concentration of HPPT groups on the surface of the CNDs. Applying two additional independent chemical and consequently optical modifications namely solution pH and annealing on the nanodots further supports our proposed explanation. Understanding the molecular origin of CND fluorescence may promote the design and control of effective CND fluorescence in optical applications.

## Abstract 2

Carbon nanodots were chemically modified to control the fluorescence and identify fluorophores.

## Graphical abstract

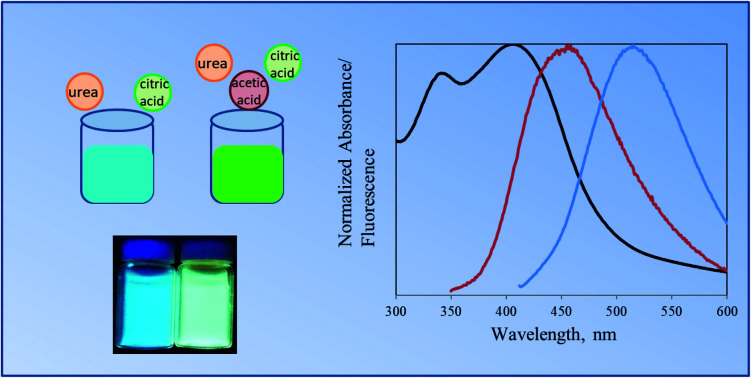

## Introduction

CNDs are partially graphitic fluorescent carbon nanomaterials.^1,2^ In recent years, they have drawn increasing scientific interest due to their valuable optical properties,^3,4^ great aqueous stability,^5^ good biocompatibility,^6^ and facile and cost-effective synthesis.^7,8^ CNDs were suggested for a wide range of electrooptic and bioimaging applications.^9–16^ Carbon dots were accidentally discovered by Xu *et al.* in 2004 during the purification of carbon nanotubes.^17^ Since then, numerous methods for carbon dot synthesis have been proposed, resulting in a wide variety of carbon nanoparticles demonstrating different chemical and optical properties.^18–23^ Importantly, doping with heteroatoms such as nitrogen, boron and sulfur was reported to drastically change the optical behavior of the nanocarbons resulting from the modification of their surfaces.^24^ Typically, CNDs are synthesized by the combined thermal reaction of a carbon source, for example citric acid (CA), and an amine precursor, for example urea (U).^19,25–30^ The resulting CNDs possess a complex chemical structure with multiple active chromophores that allow several simultaneous absorption and emission photoreactions.^22^ Moreover, the wavelength of photon emission depends on the excitation frequency. The identification of the chemical functional groups responsible for the electron transition of each photoreaction is widely discussed in the literature. However, due to their complex chemical structure, a comprehensive and defined explanation is yet to be described.^31^ Understanding of the exact chemical origin of CNDs is key for effective and controllable utilization in various applications. Optical characterization of citric acid/urea CNDs typically shows two prominent peaks in the visible rage, at 340 nm and 405 nm, responsible for the electron transition stimulating the blue and green fluorescence at 456 nm and 520 nm, respectively. The photoactivity of the 340 nm absorption peak was extensively investigated and identified.

The first reports on the reactions of urea and citric acid date back to Behrmann and Hofmann, who studied the reaction of citric acid and ammonia in 1884. They reported the formation of a blue fluorescent molecule, citrazinic acid, whose chemical structure is provided in Scheme S1 in the ESI.†^32^ In 1893, Sell and Easterfield reported the formation of citrazinic acid from the reaction between citric acid and urea at 130 °C.^33^ Schneider *et al.* confirmed the formation of citrazinic acid upon hydrothermal synthesis.^34^ Additionally, they reported a blue-shifted absorption peak caused by altering the amine precursor from ethylenediamine to hexamethylenetetramine, possibly due to further derivatization of citrazinic acid by formaldehyde. On the other hand, recently Song *et al.* suggested another fluorophore (imidazo[1,2-*a*]pyridine-7-carboxylic acid, 1,2,3,5-tetrahydro-5-oxo-, IPCA) as the true photoluminescence center that contributes the strong blue fluorescence.^35^ According to their conclusions, fluorescent graphene-like carbon nanodots containing pyrrolic, pyridinic and graphitic nitrogen are formed when citric acid amide aggregates through a condensation reaction. Thus, instead of an intermolecular reaction of citric acid amides, a ring-closing reaction to form a pyridine-based structure is likely to take place.^36^

There are two prominent absorption peaks at 340 and 405 nm. Noticeably, while most published studies investigated primarily the blue absorption (340 nm) peak, the green absorption at 405 nm and its corresponding chemical source were reported in fewer publications.^37,38^ In some studies, this peak was completely ignored. The reason for this obliviousness is that at a standard 1 : 1 amine/carbonaceous precursor molar ratio, the green emission is much weaker and hardly visible compared to the blue fluorescence. However, at higher concentrations of the amine, the green peak becomes more intense. Recent publications studying the source of this peak proposed contradicting conclusions. A study led by Kasprzyk concluded that the condensation of CA with specific β-amines results in highly fluorescent ring-fused 2-pyridones.^39^ They suggested that under aqueous conditions, the reaction between citric acid and urea leads to the formation of blue fluorescing citrazinic acid which is the final product. However, when water is allowed to further evaporate, transformation of citrazinic acid to green fluorescing HPPT (4-hydroxy-1*H*-pyrrolo[3,4-*c*]pyridine-1,3,6(2*H*,5*H*)-trione, whose chemical structure can be found in Scheme S1†) becomes dominant. Demchenko *et al.* associated the shoulder at 430 nm with π–π-stacked H-type aggregates.^40^ Thus, the exact photoactivity of synthesized carbon nanodots is still unclear and a proper explanation is required. This year, Strauss *et al.* succeeded in separating CND solution into two different particles by column chromatography, proving that the blue and green fluorescence originates from two different materials.^10^ It is therefore essential to identify the different surface compositions that lead to the two different fluorescent colors.

This study is aimed at clarifying the origin of the two prominent absorption peaks and their corresponding fluorescence. In order to better understand the chemical compositions responsible for each optical transition, we examined the optical behavior upon various chemical modifications of CNDs. First, different molar ratios of the two precursors, urea and citric acid (U/CA), during the thermochemical preparation of CNDs were investigated. We also added a third carbonaceous precursor to the synthesis process, namely acetic acid (AA), and compared the resulting photoactivities. Being a simple carboxylic acid, AA is a fragment of citric acid. Therefore, an investigation of its impact on the product may reveal the exact role of citric acid in the reaction mechanism. In addition, strong evidence for the chemical composition/photoactivity correlation was found in the acidity of the dispersing solvents and various surface modifications. Joint observations from optical experiments and chemical analysis of surface-modified CNDs shed light on the chemical origins of multi-photoactivity.

## Results and discussion

### (1) Physical characterization

**Fig. 1:**
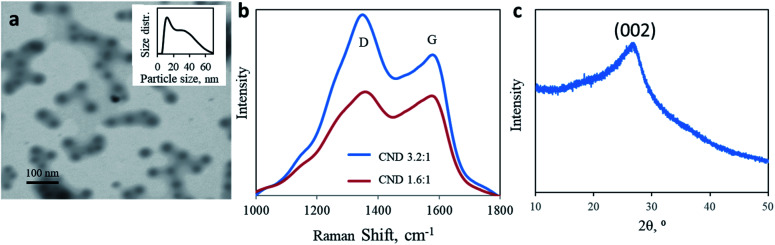
Physical analysis of CNDs. (a) High magnification STEM image of 3.2 : 1 CNDs mag. 250 kx. Inset: particle size distribution obtained with DLS. (b) Raman spectra of 3.2 : 1 (blue) and 1.6 : 1 (red) CNDs obtained upon excitation at 532 nm. (c) Typical pXRD pattern of CNDs.

To reveal the origin of the two electronic transitions and the two corresponding optical absorptions/emissions, CNDs were synthesized using two different molar ratios of urea and citric acid: 1.6 : 1 ratio, similar to the standard 1 : 1 ratio used in most studies, and 3.2 : 1 U/CA CNDs. A microwave-assisted hydrothermal reaction results in a dark brown residue due to a carbonization reaction. Both products are highly dispersible in water. The nanoparticles adopt a sphere shape with a size distribution of 10–60 nm. The predominant diameter is around 10 nm, with a tail up to 70 nm (mean diameter = 15.73 nm, Fig. 1a, in good agreement with AFM measurements shown in Fig. S1†). Raman spectroscopy of both CNDs supports the complexed chemical structure of the nanoparticles (Fig. 1b). Two prominent peaks are observed at 1350 and 1582 cm^−1^, corresponding to D and G bands, respectively, attributed to the presence of sp^3^ and sp^2^ hybridized carbon–carbon bonds typically found in carbonaceous materials. The ratio between the intensities of D and G bands allows quantification of the graphitization level of nanocarbons. Changing the molar ratio from 1.6 : 1 to 3.2 : 1 increases the *I*_D_/*I*_G_ ratio from 1.035 to 1.309 (Fig. 1b). A higher urea content results in lower graphitization. The partially graphitic crystallinity is further confirmed by X-ray diffraction (XRD). Both products were characterized by a broad peak centered at 2*θ* = 26° (Fig. 1c). The XRD patterns of both 1.6 : 1 and 3.2 : 1 CNDs were similar. The thermal stability of the products, up to 400 ° C, supports their partial graphitic nature (ESI Fig. S2†).

### (2) Optical properties

**Fig. 2:**
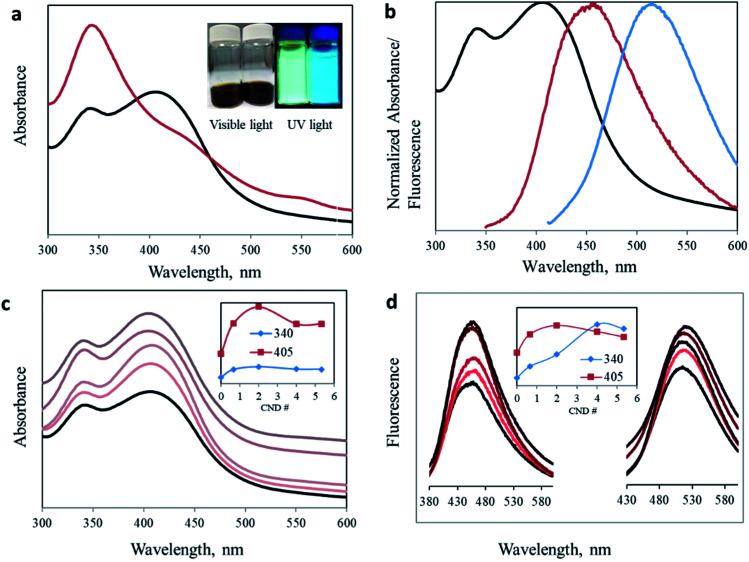
Optical characterization: (a) UV-Vis spectra of CNDs with urea/citric acid molar ratios of 3.2 : 1 (black) and 1.6 : 1 (red). Inset: CND dispersion under visible and UV light. Molar ratios 3.2 : 1 (left) and 1.6 : 1 (right). (b) CND 3.2 : 1 optical performance. Normalized absorption (black) and photoluminescence emission spectra (*λ*_ex_ = 340 nm (red) and 405 nm (blue)). (c) UV-Vis spectra of CND 0 (black) and CND 0.5–5 (light to dark). Inset: maximum absorbance intensity *versus* AA ratio dependence. (d) Fluorescence spectra of CND 0 (black) and with different AA ratios (*λ*_ex_ = 340 nm (left) and 405 nm (right)).

One of the main advantageous properties of carbon nanodots is their bright photoluminescence. Fig. 2a presents the considerable effect of the proportion altering of the organic precursors on the optical properties of the produced CNDs (deconvolution of the overlapping peaks is presented in Fig. S3†). The traditional 1 : 1 ratio results in one prominent absorption peak at 340 nm. However, by changing the ratio of the precursors, we managed to significantly enhance the intensity of the secondary 405 nm peak. Surprisingly, the green peak at 405 nm no longer appeared as a minor shoulder seen in most publications, but rather as an intense blue absorption peak (*I*_340_/*I*_405_ = 0.844, see Table 1). Moreover, the change is noticeable under both visible and UV light. The aqueous dispersion turns from a dark brown (1.6 : 1) to an orange solution (3.2 : 1) (Fig. 2a inset). An inclusive optical examination was conducted to evaluate the optical properties of these two synthesized carbon nanodots. Fig. 2b presents the normalized absorbance and fluorescence spectra at excitation wavelengths of 340 and 405 nm for CNDs with a 3.2 : 1 urea/citric acid molar ratio.

**Table 1:** Dependence of maximum absorption intensity on the urea : citric acid : acetic acid molar ratio

Sample	U : AC : AA ratio	*I*_340_ max	*I*_405_ max	*I*_340_/*I*_405_
CND 0	3.2 : 1 : 0	0.315	0.373	0.844
CND 0.5	3.2 : 1 : 0.5	0.334	0.448	0.745
CND 2	3.2 : 1 : 2	0.341	0.489	0.697
CND 4	3.2 : 1 : 4	0.335	0.448	0.748
CND 5	3.2 : 1 : 5	0.335	0.447	0.749

To reveal the chemical configuration responsible for the intense peak at 405 nm, we modified the chemical structure of the CNDs by adding another precursor, namely acetic acid (AA). This additional carboxylic source was added in different molar ratios to a constant 3.2 : 1 urea/citric acid aqueous mixture. These new acetic acid modified CNDs will henceforth be described according to the molar ratio of acetic acid added to 3.2 : 1 U/CA. For example, sample CND 2 is the CND synthesized from 3.2 : 1 : 2 urea/citric acid/acetic acid mixtures. A comprehensive optical characterization of carbon nanodots was performed by using UV-visible, fluorescence and photoluminescence spectroscopy. Fig. 2c shows the UV-visible spectra of the synthesized CNDs with different U : CA : AA ratios. Two distinct peaks at 340 nm and 405 nm were obtained in all the samples. Importantly, acetic acid addition of only half of the citric acid increases the intensity for both peaks, confirming the effect of the acetate ion on the surface of carbon nanodots. However, the effect is not identical for all samples. In samples where small amounts of AA were added, for example CND 0.5, the intensity increase of the 405 nm peak is more pronounced than for the 340 nm peak (Table 1). This trend continues as the AA concentration increases until it stops at CND 2. This is possibly because of the low boiling point of AA, such that at too high concentrations, a large portion is evaporated without effectively participating in the synthesis process. In addition, when AA is in excess, two molecules of AA undergo a condensation reaction to form acetic anhydride, (CH_3_CO)_2_O. The reacted AA cannot any longer participate in the reaction we proposed to form the keto-HPPT. The acetic anhydride or similar products can become part of the carbon nanodots but they do not demonstrate optical properties and cannot be removed during the washing of CNDs.

The fluorescence of CNDs with different AA ratios was measured at two excitation wavelengths, namely 340 and 405 nm, corresponding to the two reported absorption peaks (Fig. 2d). For both excitation wavelengths, addition of acetic acid resulted in enhanced fluorescence. The most effective ratio was in good agreement with that of the strongest recorded absorption. Again, this increase is valid up to a certain ratio, after which a higher AA concentration leads to a reduced optical effect (Fig. 2d inset, and Table S1 and Fig. S4†).

Nevertheless, the increase of the 405 nm peak as a result of AA addition in the synthesis of the CNDs is the opposite of the anticipated result. We have shown that higher concentrations of urea result in an increased intensity of the 405 nm peak. For a reasonable assumption, we may attribute it assumedly to the most significant change, the higher nitrogenous groups on the surface of the nanoparticles. Therefore, it is surprising that the addition of a nitrogen-free molecule such as AA imposes the same trend. Therefore, a thorough chemical analysis of the different products is required.

### (3) Chemical analysis

**Fig. 3:**
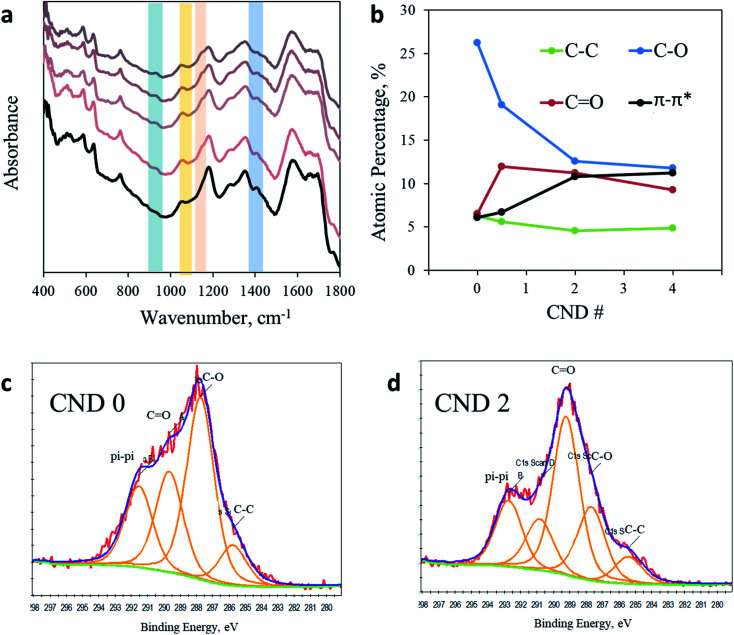
Chemical analysis. (a) FTIR spectra of CND 0 (black line) and CND 0.5–5 (light to dark) in the region from 400 cm^−1^ to 1800 cm^−1^. (b) Quantification of the C 1s XPS response in CND 0–4. (c) C 1s spectra of CND 0. Fitting was performed according to Table S3.† (d) C 1s spectra of CND 2 with fitting to different carbon bonds.

Puzzled by this surprising photochemical observation, we conducted a thorough chemical analysis to understand the impact of the AA addition. Fig. 3a shows the FTIR spectra of CNDs synthesized with a varying ratio of AA. A more detailed analysis of the full FTIR spectra can be found in the ESI in Fig. S5.† The region from 1800 to 4000 cm^−1^ shows no noteworthy changes upon AA addition. However, noticeable impacts are observed between 400 cm^−1^ and 1800 cm^−1^. For example, the prominent peak at 1400 cm^−1^ arising from a CH_3_ bending vibration clearly observed in AA-free CNDs gradually decreased as more AA was added into the sample, until nearly complete elimination in CND 5. In contrast, the peaks at 1150 cm^−1^ and 1056 cm^−1^, originating from C–O or C–N and C–O–C stretching, respectively, are observed in all samples containing AA but not in CND 0. In addition, the peak at 925 cm^−1^ arising from unsaturated sp^2^ C–H(C=C–H) bending vibrations is present in all samples containing AA, but is completely absent in sample CND 0. In conclusion, FTIR analysis reveals that AA addition forms CNDs with a higher oxidation state. These nanoparticles contain fewer methyl groups and more oxidized and sp^2^ carbons. Zeta potential measurements support this conclusion, showing a similar trend. While CND 0 displays a small charged surface (three measurements mean 12.8 mV), CNDs 0.5–5 demonstrate increasingly negative zeta potentials graduating in the range of 15.3–24.5 mV for CND 2 (Fig. S6†).

Since the photoactivity of CNDs occurs on the surface of the nanoparticles, a better characterization of the surface is of great importance. X-ray photoelectron spectroscopy (XPS) was employed in order to analyze the chemical composition of CND 0–4 (full spectra in Fig. S7 in the ESI†). While the ratio between elements C, O, and N remains similar in all samples, examination of the C 1s peak shows clear evidence for the chemical modification imposed by AA addition (Fig. S8 and Table S3 in the ESI;† the C 1s peak was fitted according to the position key presented in Table S4†). The dominant peak in CND 0 is attributed to C–O (or C–N) bonds at 286.5 eV, occupying 26.5% of the total carbon peak area. However, this peak is significantly reduced in CND 0.5–4, down to 17.5, 12.7 and 12.2%, respectively. The sharp drop in C–O intensity is compensated by an increase of the C=O, C–O–C and π–π* response (Table S3†). Particularly, the C=O peak at approximately 290 eV increases from 6.5% in CND 0 to 11.95, 11.25 and 9.20% in CNDs 0.5–4 (Fig. 3c and d). In light of these results we can conclude that the peak at 1150 cm^−1^ obtained in FTIR should be attributed not to C–O but to C–N stretching. These changes are accompanied by nearly doubling of the aromatic carbon (π–π*) interactions in the CNDs from 6% in CND 0 to 11.5% in CND 4. The XPS results are in good agreement with the FTIR data described above, pointing to a higher oxidation level of the surface of the CNDs. Specifically, more double bonds between carbon and oxygen atoms and higher aromatization (more rings) were recorded. There is no significant shift in the N 1s position or area in all samples, located between 402.79 and 403.80 (Fig. S9†).

The chemical analysis described herein allows a reasonable explanation for the effect of AA addition on the optical properties of CNDs. It is well established that surface functional groups play an important role in determining the absorption wavelengths of carbon nanodots.^41–44^ In most of the previous publications, when a small amount of urea is used in the precursor mixture, the dominant peak at 340 nm is reported to originate from the n–π* transition of C=O bonds.^34,45,46^ In that case, only a minor shoulder is observed at 405 nm. However, a clear correlation is provided between the amount of urea in the precursor mixture and the intensity of the 405 nm absorption and corresponding 520 nm emission peaks. This observation was reported previously in a publication by Dam *et al.*^47^ Urea is the only source for nitrogenous compounds during the formation of the CNDs and is thus the source for nitrogen-heterocycle formation. Ultimately, pyridinic groups and their derivatives cause the intense electronic transition at 405 nm. At the same time, as more pyridine groups are present on the surface of the CNDs, the intensity of the 340 nm absorption is quenched after some of the excited electrons are channeled toward the less energetic transition. This also explains the intensity lowering of the blue fluorescence in samples containing higher concentrations of urea we have shown in the first part (Fig. 2a). Consequently, with an increasing ratio of urea, a comparably more intense peak appeared at 405 nm in the absorption spectra.

**Scheme 1:**
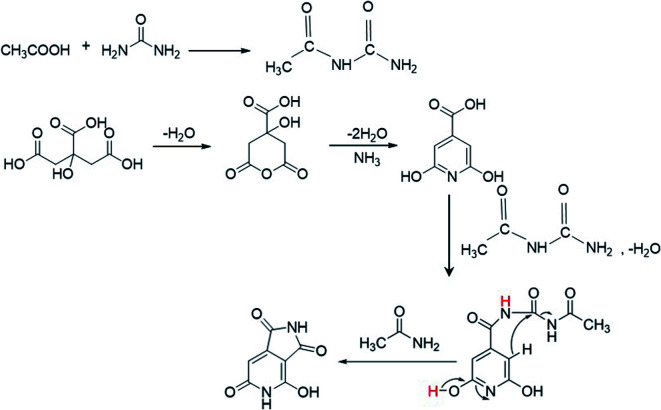
The proposed mechanism of HPPT formation in the presence of AA during the synthesis process.

The XPS data presented above further assist in revealing the chemical state responsible for the second peak at 405 nm. According to the XPS data, as more AA is added in the synthesis of the CNDs, more oxygen-related carbonyl groups are formed on the surface. Moreover, a reduction of C–O group content and increase of cyclic carbons (π-rings) were recorded in XPS data of samples with AA. These results may help in solving the question raised above regarding the surprising increase in the pyridinic transition that is observed upon nitrogen-free AA addition. Acetic acid first reacts with urea to form a derivative of acetamide named *N*-carbamoylacetamide.^48,49^ The formation of this intermediate from urea and AA was proved by NMR (Fig. S10†). In course of the reaction it undergoes a secondary reaction with citrazinic acid by a condensation reaction to form an intermediate molecule that subsequently forms the keto isomer of HPPT. The proposed mechanism is illustrated in Scheme 1. The ammonia released upon dehydration of urea is condensed with the cyclized six membered ring of citric acid to form citrazinic acid. Further derivatization of this fluorophore becomes possible in the presence of AA. In a multi-step reaction, AA forms an intermediate molecule that later participates in a different paths of formation of the fluorophore HPPT (see Scheme 1; for a more detailed description including the electron pathway see the ESI in Scheme S2†). This mechanism is in agreement with the proposal made by Kasprzyk *et al.* regarding the formation of the species responsible for the 405 nm peak. They deciphered that the peak at 405 nm originated from the fluorophore HPPT, which is formed after removal of water from the citrazinic acid intermediate.^39^ We suggest that in the absence of AA, two isomers of HPPT are formed equally, while only one demonstrates optical activity. Importantly, the results of our alternative path involving AA prevent the equilibrium of the keto with the enol as the final products. The only final product formed is the keto isomer of HPPT. Therefore, XPS measurements showed lower amounts of C–O and higher amounts of C=O groups on the surface. The keto isomer is the species responsible for the 405 nm peak. Therefore, addition of AA results in stronger absorption at this wavelength. To conclude, our study points out that the 340 nm peak arises from the n–π* bond in citrazinic acid, while the 405 nm absorption peak originates from the 2-pyridone ring (NHC=O group) of the six membered ring in the HPPT molecule.

### (4) pH dependence

**Fig. 4:**
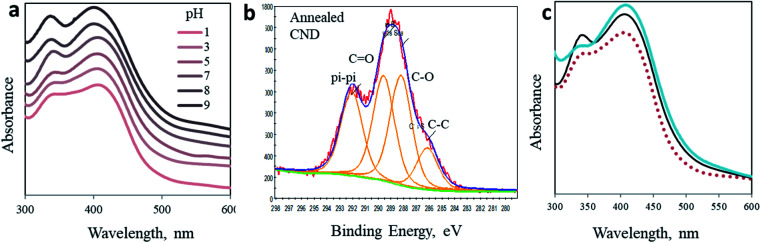
Modified CNDs. (a) pH dependence of absorption spectra of CND 0. (b) C 1s XPS spectra of the annealed CNDs. (c) Absorption spectra of the as-prepared CND 0 (black) and CND 0 after thermal annealing (blue). For comparison, the absorption spectrum in acidic solution is presented as a dashed red line.

To embrace our conclusion, we reviewed the photoactivity of CND 0 in media of varying acidity, stimulating comparable effects to surface chemical modifications. Interestingly, no shift in the position of both peaks is observed (Fig. 4a). Yet, the shape of the spectra and the ratio between the two peaks change significantly. These changes may indicate the chemical origin of the photoreactions in CNDs. In acidic solutions, the 340 nm peak is depressed as the pH decreases until it is almost eliminated at pH 1 and, naturally, the fluorescence spectra change accordingly (Fig. S11†). On the other hand, in basic solutions this peak becomes sharper and is equal to the 405 nm peak (see Table S2†). At the same time, the 405 nm peak is hardly affected. These results can be explained by the fact that under acidic conditions the surface carboxylate groups, which are responsible for the 340 nm peak, are very prone to protonation.^50^ Ultimately the concentration of the C=O double bond is lowered, subsequently decreasing n–π* transitions. The reverse phenomena occur in a basic environment. Deprotonation processes of carboxylate moieties on the surface of the nanoparticles induce a stronger resonance within the O–C=O bond of citrazinic acid. This in turn increases the related electronic population of this state and electron transition upon light irradiation. The fluorescence of the AA added samples in different pH follows the same trend (Fig. S12†).

To ensure that the changes in photoactivity originate solely from the pH environment and not from the constant chemical change caused by the solvent acidity, we changed the pH of the solvent with the CNDs dispersed in it from basic to neutral and acidic solutions. The absorption spectra showed complete recovery resuming the exact shape of the spectra related to CND in neutralized pH (Fig. S13†). The shape of absorption spectra at pH 8 after neutralizing was identical to that of the spectra of the CNDs that were dissolved originally in pH 7 solution. Likewise, the spectra of the same dispersion after acidifying it down to pH 1 show the same pattern as that of the initial pH 1 dispersion. The full recovery of the photoactivity of the CNDs after dispersion under harsh basic and acidic conditions also points to the high chemical stability of the nanoparticles.

### (5) Thermal annealing

Finally, we simulate the chemical impact of C=O elimination from the surface of the nanoparticles by annealing. The CND 0 samples were heated up to 250 °C under an inert atmosphere for 3 hours. As a result, the chemical composition of the nanoparticles was modified, as revealed in XPS experiments (Fig. 4b and ESI Fig. S14 and S15†). Ultimately, the UV-Vis spectrum of the annealed particles also changed (Fig. 4c). A comparison of the absorption spectrum of annealed CNDs to that of CNDs in pH 3 solution offers three independent observations of the optical behavior of CNDs. In accordance to the above prediction, the 340 nm peak is flattened as a result of the elimination of C=O moieties. The shape of the annealed particles is remarkably similar to that recorded in acidic solution (pH 3, dashed curve in Fig. 4b). This similarity confirms the chemical effect of C=O elimination on the 340 nm peak.

## Conclusions

In this study, we investigated the chemical species responsible for producing the bright fluorescence of urea/citric acid CNDs. Different ratios of organic precursors generate different intensities of optical peaks. Specifically, a remarkably enhanced green absorption peak at 405 nm was clearly observed at a 3.2 : 1 molar ratio although it is hardly visible in a standard 1 : 1 ratio sample. Elucidation of the observation was achieved by examining chemically modified CNDs. A third organic molecule, acetic acid, was added in the synthesis in different concentrations. The addition of AA imposed dramatic effects on the CND spectra. Spectroscopic analysis of the products revealed that the strong absorption at 340 nm occurs in samples where high amounts of C=O groups are found on the surface, while absorption at 405 nm increases in samples where more pyridine rings are present. The amount of C=O and pyridinic chromophores on the surface is affected by the addition of AA. In the proposed chemical mechanism, a product of the reaction between acetic acid and urea participates in a thermal condensation reaction with citrazinic acid to form HPPT, a molecule known to generate the 405 nm absorption. To further relate the green peak to pyridine rings, we studied the optical activity dependence in solutions of varying acidity. The spectra obtained in solutions of varying acidity/basicity showed similar effects imposed by AA addition. Particularly, the 340 nm peak was enhanced in basic solutions while it was significantly depressed in acidic environments. Finally, the carbon nanoparticles were annealed to remove oxygen from their surface. Predictably, the 340 nm absorption peak of the annealed CNDs decreases, resembling the effect in acidic pH (pH 3). With the understanding of the exact chemical origin of CNDs, and controlling their fluorescent properties, applications utilizing CNDs can be designed more effectively.

## Experimental

### Materials and instrumentation

All chemicals, including urea, citric acid and acetic acid, were purchased from a commercial supplier and used without further purification. UV-Vis absorption spectra were recorded on a Varian Cary 50 Bio UV-Vis spectrophotometer. Fluorescence spectra were recorded on a Varian Cary Eclipse fluorescence spectrophotometer. All peak separation and curve fitting procedures were performed using OriginPro 2020. Raman spectra were collected with an XploRA ONE™ micro-Raman system (Horiba Scientific, Japan) using a Kratos AXIS-HA spectrometer with 532 nm laser power, using a monochromatized Al Kα source. Fourier transform infrared (FTIR) spectra were measured using a Bruker Vertex 70. XPS spectra were recorded using a Kratos Axis Ultra DLD spectrometer equipped with a monochromatic Al Kα X-ray source (*hn* 1/4 1486.6 eV). High-resolution spectra were calibrated using carbon tape (Ted Pella) with a known C 1s binding energy of 284.6 eV. Raw data were processed using Casa XPS software (version 2.3.16). C 1s spectra were fit using Gaussian–Lorentzian line-shapes for all spectral components except for the sp^2^ C–C component, which was fit with an asymmetric line. Particle size was determined with a Nanophox dynamic light scattering (DLS) sensor by Sympatec, Germany. STEM images were produced using an ultra-high resolution Tescan FE-SEM (Czech Republic).

### CND synthesis

Previously documented protocols were employed for the CND synthesis. In a typical synthesis, citric acid (0.5 g) and urea (0.5 g) were dissolved in double distilled water (2 ml) as precursors for the preparation of CNDs with a urea/citric acid molar ratio of 3.2 : 1. In another case, citric acid (1 g) and urea (0.5 g) were added to double distilled water (2 ml) to prepare CNDs having a urea/citric acid molar ratio of 1.6 : 1. A beaker with the reaction mixture was heated in a domestic microwave (700 W) until the solvent was evaporated. After the beaker was cooled to room temperature, acetone was added to it and then the whole reaction mixture was centrifuged at 4000 rpm for 15 minutes to separate the large agglomerated particles. Then the mixture was filtered. Washing was repeatedly done with acetone followed by drying for a few hours. This solid residue was then dissolved in water for further measurements. Although the reactions were conducted at different scales, they yielded the same products. In all experiments, the ratio between citric acid and urea was kept constant.

The annealed CNDs were thermolyzed at 250 °C for 3 hours in a tube furnace. The reaction vessel made of alumina, containing the as-synthesized CNDs, was placed in the center of the tube furnace. The furnace was heated to the final temperature with a heating rate of 6 K min^−1^ and kept at the final temperature for 2 h. A gas flow of nitrogen was used to remove gaseous reaction products.

A study of the response of CNDs to different pH was carried out by adding solid samples of CNDs directly into solutions of various pH prepared by adding HCl to make the solutions acidic and NaOH to make them basic, monitored using a pH meter. All the measurements were performed in triplicate.

### Optical characterization

#### UV-Vis and fluorescence measurements

2 mg of the solid brown residue of CNDs obtained after centrifugation followed by filtration and drying was dissolved in 5 ml of double distilled water. 250 μl of the CND aqueous solution was then added to a 3 ml cuvette and topped up with water and the UV-Vis and fluorescence spectra were recorded. All the samples were filtered through 0.22 μm membrane syringe filters to remove all the large particles from the solution before taking the sample for UV-Vis and fluorescence analysis.

## Conflicts of interest

There are no conflicts to declare.

## Supplementary Material

NA-003-D0NA00871K-s001
