# Supplementary material for: Surface modifications of carbon nanodots reveal the chemical source of their bright fluorescence
Source: Nanoscale Adv. 2020 Dec 10;3(3):716–24. doi: 10.1039/d0na00871k (PMC9417464; doi:10.1039/d0na00871k)
Supplement: NA-003-D0NA00871K-s001 [file NA-003-D0NA00871K-s001.pdf]

Electronic Supplementary Material (ESI) for Nanoscale Advances.
This journal is © The Royal Society of Chemistry 2020

Supporting information to

Surface modifications of carbon nanodots reveal the chemical source of their bright fluorescence

Asmita Dutta[1], Shimon T. Y. Trolles-Cavalcante[1], Annie Cleetus[1], Vered Marks[1], Alex Schechter[1], Richard D. Webster[2], Arie Borenstein*[,1]

1- Department of Chemical Sciences, Ariel University, Ariel, Israel. Email: arieb@ariel.ac.il
2- Division of Chemistry & Biological Chemistry, School of Physical and Mathematical Sciences, Nanyang Technological University, Singapore, 637371

Table of Contents

Tables 3

Photoactivity 3

Table S1. 3

Table S2. 4

X-ray Photoelectron Spectroscopy 5

Table S3 5

Table S4 5

Figures and Schemes 6

Chemical Structure 6

Scheme S1. 6

Atomic Force Microscopy (AFM) 7

Figure S1: 7

Thermal Gravimetric Analysis (TGA) 8

Figure S2. 8

Photo activity 9

Figure S3. 9

Figure S4. 10

Fourier Transform Infra-Red Spectroscopy (FTIR) 11

Figure S5. 11

Zeta Potential 12

Figure S6. 12

X-ray photoelectron spectroscopy (XPS) 13

Figure S7. 13

Figure S8. 13

Figure S9. 14

NMR of N-carbamoyl acetamide 15

Figure S10 15

Optical behavior of CND 0-5 in solutions with different pH. 16

Figure S11. 16

Figure S12. 17

Recovery of optical behavior after neutralizing acidity of solvent 18

Figure S13. 18

XPS for annealed CND 19

Figure S14. 19

Figure S15. 19

Acetic acid assisted HPPT formation mechanism 20

Scheme S2. 20

Tables

Photoactivity

Table S1.Maximum fluorescence intensities of CND 0-5 in neutral water. The data was extracted from **Figure 2d**.

Sample	U:CA:AA ratio	I_{340}	I_{405}	I_{340} / I_{405}
CND 0	3:1:0	325.49	405.72	0.802
CND 0.5	3:1:0.5	362.41	464.76	0.779
CND 2	3:1:2	400.93	492.57	0.813
CND 4	3:1:4	496.06	473.01	1.048
CND 5	3:1:5	482.99	455.78	1.059

Table S2. Maximum absorption intensities of CND 0 in aqueous solutions with different pH. The data was extracted from **Figure 4a**.

Solution Sample	I_{340}	I_{405}	I_{340}/I_{405}
pH 1	0.316	0.343	0.921
pH 3	0.288	0.338	0.852
pH 5	0.295	0.365	0.810
pH 8	0.341	0.396	0.860
pH 9	0.394	0.429	0.919

X-ray Photoelectron Spectroscopy

Table S3. Atomic percentage extracted from XPS data of C1s, O1s, N1s and their calculated fittings

**CND #**	**0**	**0.5**	**2**	**4**	**5**
C-C	6.25	5.6	4.57	4.86	7.48
C-O	26.22	19.06	12.57	11.77	19.79
C-O-C	15.63	18.15	22.69	25.19	18.96
C=O/ COOH	6.49	11.94	11.25	9.25	5.14
pi-pi	6.10	6.71	10.78	11.22	10.14
C total	60.44	61.46	61.86	62.29	61.51
O	21.93	21.95	20.9	20.9	20.47
N	15.39	16.58	16.75	16.75	18.15

Table S4. XPS C 1s Spectra Fitting in **Figure 3c,d and 4c.**

sp^2	sp^3	C-O	C-O-C	C=O	COOH	pi-pi*
284.6	285.4	286.5	287.62	288	288.7	290.99

* All units in eV.

Figures and Schemes

Chemical Structure

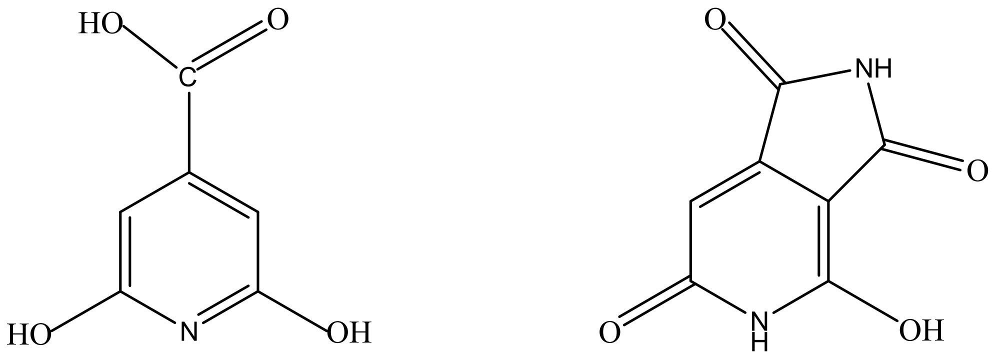

Scheme S1.Chemical structure of Citrazinic acid (left) and HPPT (Right)

Atomic Force Microscopy (AFM)

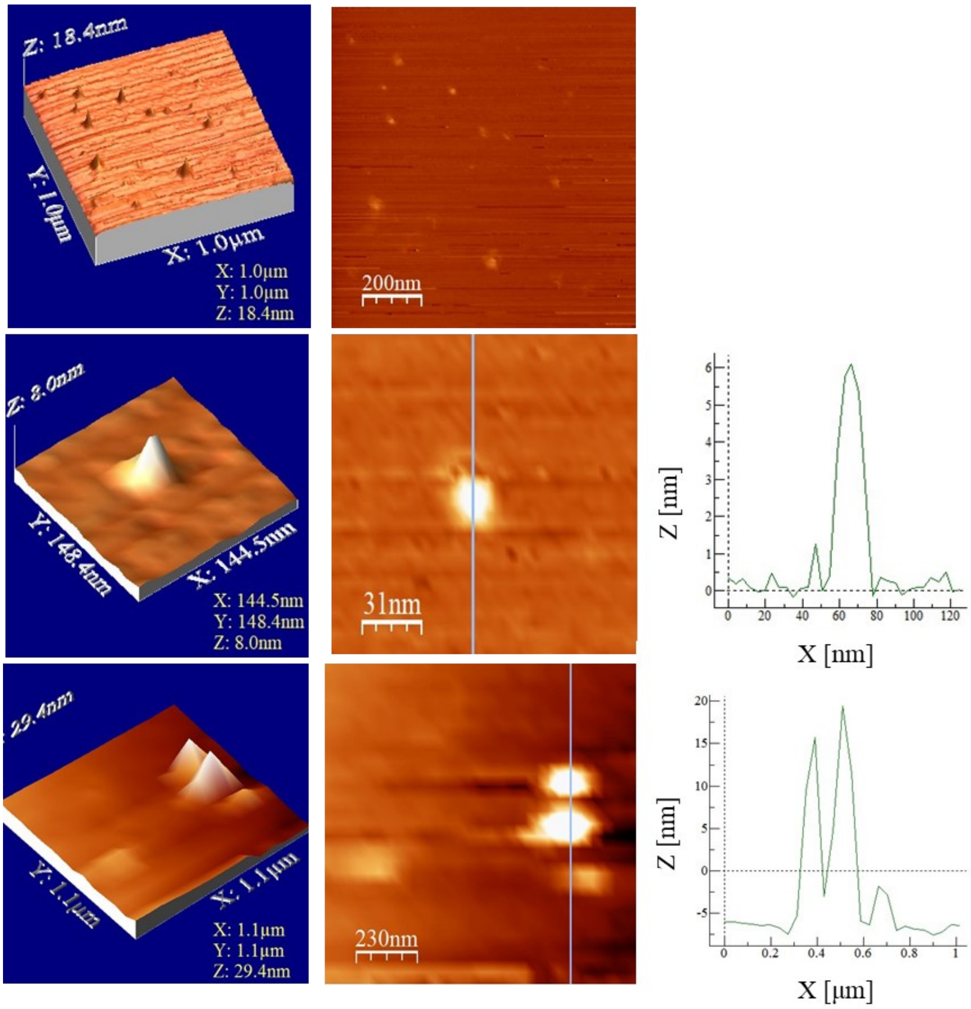

Figure S1: Atomic force microscopy (AFM) for a typical CND0. In each raw the left column shows 3D topology of the samples, middle column show 2D micrograph with color representing height (brighter=higher) and right column show X-Z plot of the cross-line in the middle column. The particles show spherical shape with mean Z-dimensions of 12 nm, in good agreement with DLS measurement.

Thermal Gravimetric Analysis (TGA)

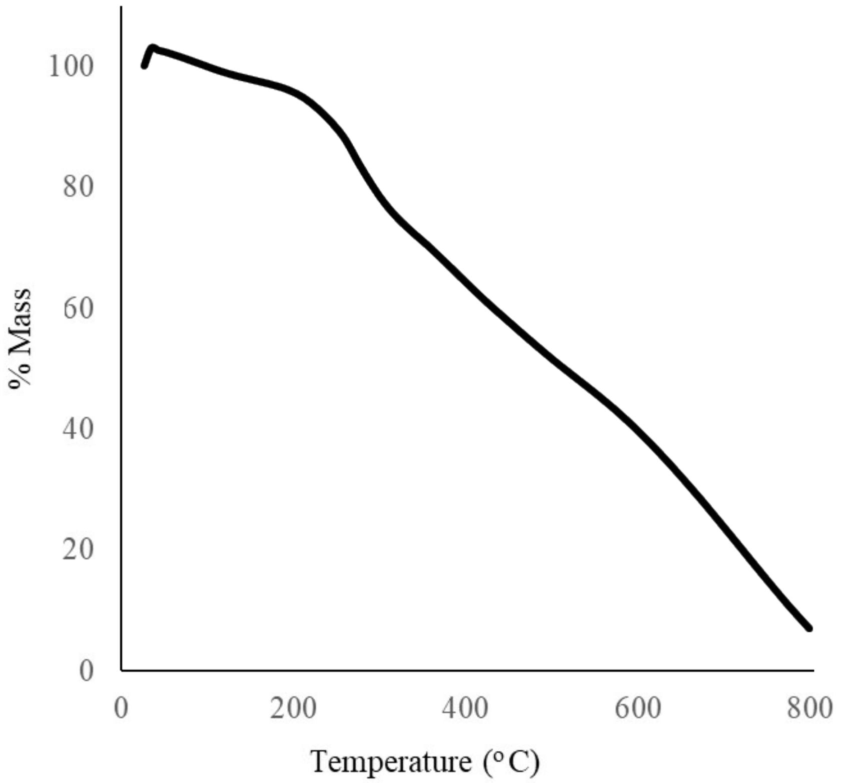

Figure S2.Thermal Gravimetric Analysis (TGA) of CND 0. The thermal stability was measured while the sample was exposed to air. The mass loss of less than 50% up to 450^{0}C indicates high thermal stability for carbonaceous materials and imply the graphitic nature of the nanoparticles.

Photo activity

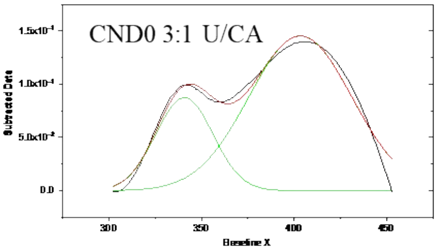

Area Intg	FWHM	Max Height	Center Grv ty
3.36484	36.408	0.08736	340.77868
9.7549	65.48494	0.14536	403.37577

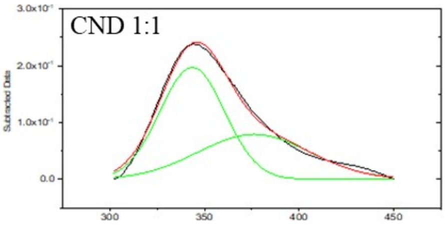

Area Intng	FWHM	Max Height	Center Grvty
8.40242	40.43025	0.19677	343.57335
5.77854	69.62559	0.07894	376.21399

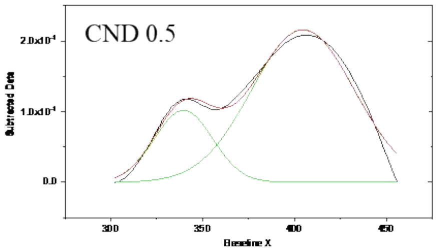

Area Intg	FWHM	Max Height	Center Grv ty
4.01164	37.3307	0.10186	339.59975
14.61771	65.79905	0.21586	404.57016

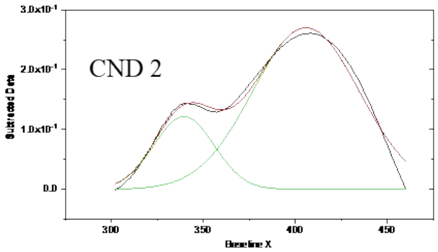

Area Intg	FWHM	Max Height	Center Grv ty
4.93559	38.54001	0.12167	339.44345
19.00491	68.18402	0.27008	405.72399

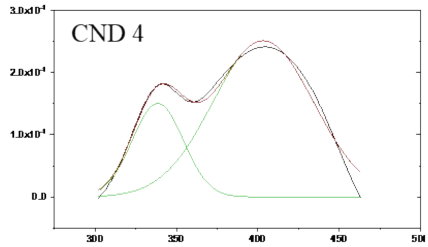

Area Intg	FWHM	Max Height	Center Grv ty
5.85077	36.9065	0.15044	338.49904
18.97313	72.89274	0.25142	403.25117

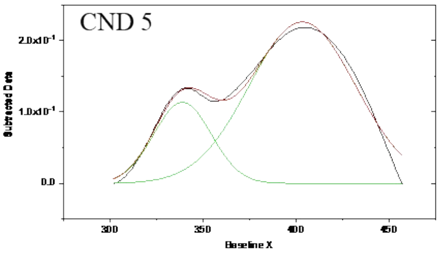

Area Intg	FWHM	Max Height	Center Grv ty
4.35419	36.40673	0.1133	339.00158
15.65706	67.18764	0.22576	403.36169

Figure S3. Peak deconvolution and data for absorption peaks of different CNDs.

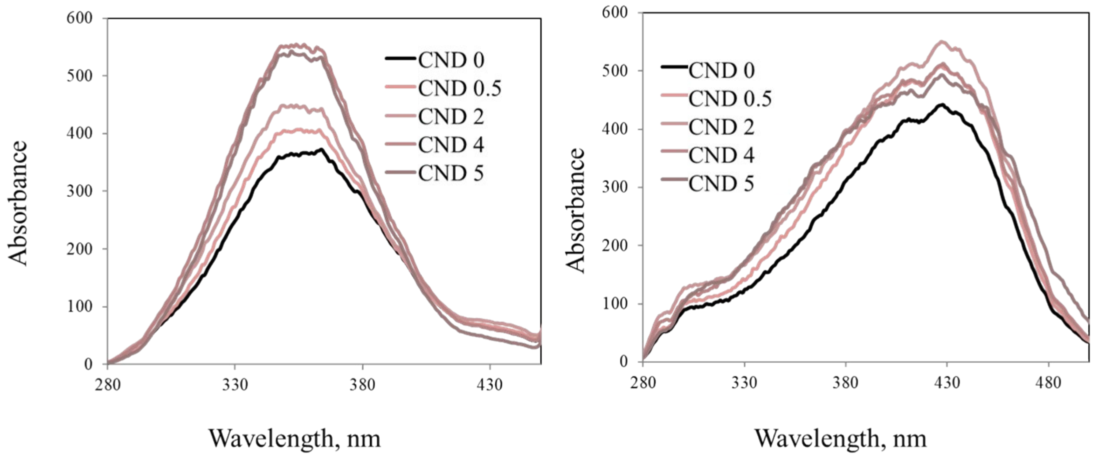

Figure S4.Photoluminescence excitation (PLE) spectra for CNDs 0-5 upon emission wavelength of 456 nm (Left) and 520 nm (Right).

Fourier Transform Infra-Red Spectroscopy (FTIR)

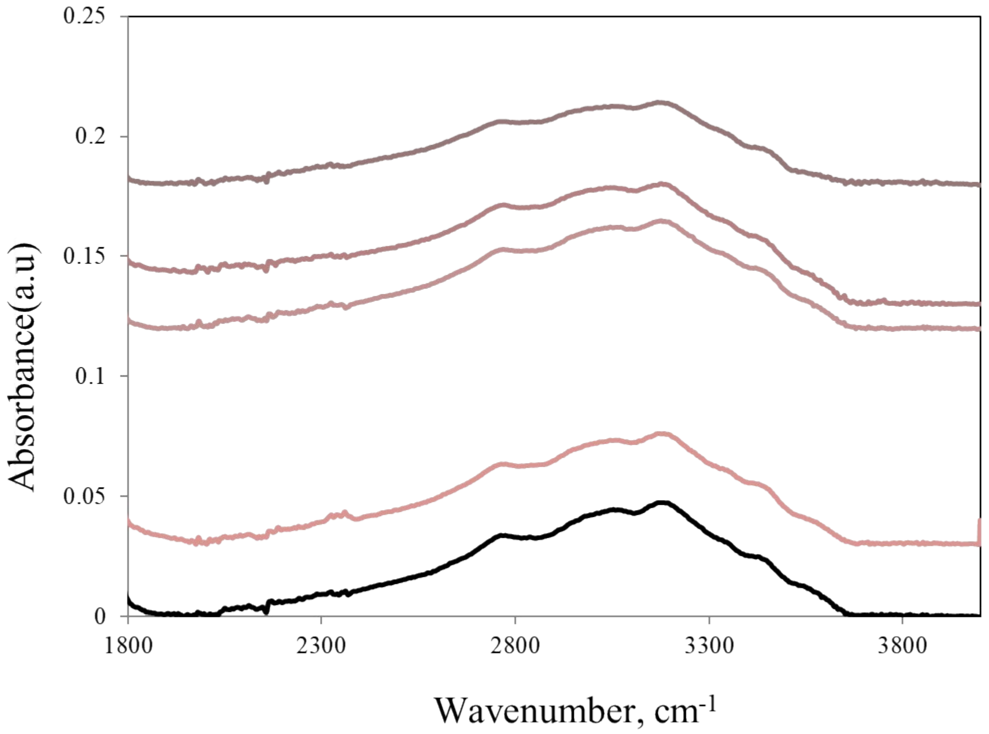

Figure S5.Complementary spectra of FTIR of CND 0-5 1800-4000 cm^{-1}.

Typical broad absorption features around 3200 cm^{-1}, confirming the presence of alcohol (-OH) and amine (NH_2) in all samples. The peak at 3000 cm^{-1} is attributed to sp^2 C-H stretching of methyl. Another peak at 2700 cm^{-1} is generated due to stretching of C-H bond of sp^3 type molecule ($-CH_3$, $-CH_2$).

Additionally, the peak at 1651cm^{-1} is attributed to carbonyl group (–C=O stretching). Moreover, the nearby peak at 1710 cm^{-1} signifies α,β unsaturated carboxylic acid, increases upon increasing AA addition. The peak at 1560 cm^{-1}, originated from N-H bending vibration. Another small peak at 650 cm^{-1} is due to sp^2 C-H bending.

Zeta Potential

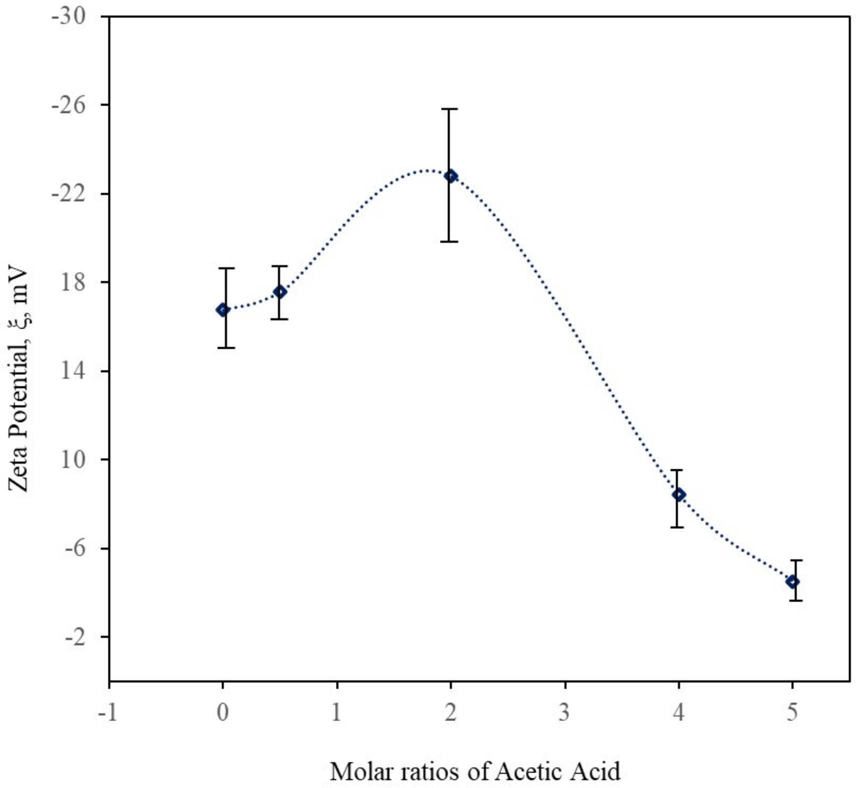

Figure S6. Zeta potentials of CND 0-5 measured in neutral aqueous solution.

X-ray photoelectron spectroscopy (XPS)

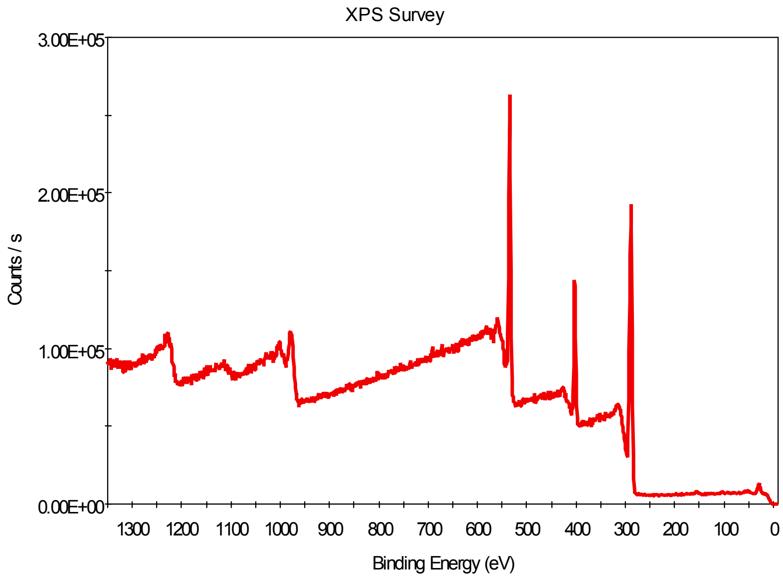

Figure S7.Full XPS spectra of CND 0.

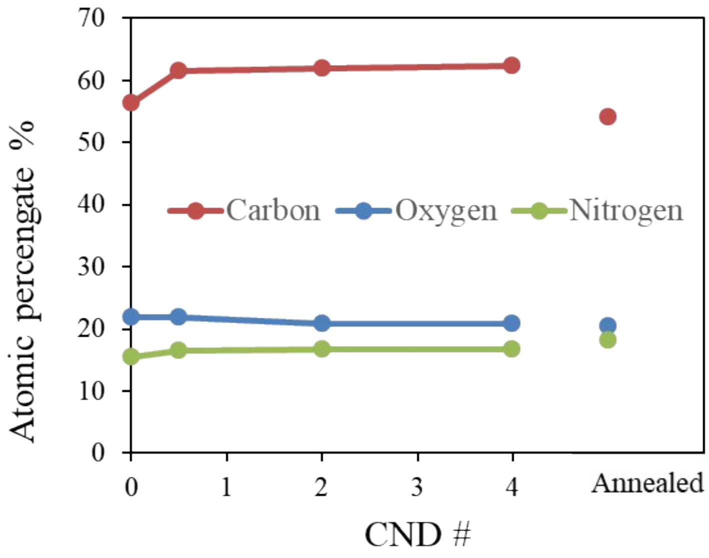

Figure S8. Elemental analysis recorded by XPS measurement in CND 0-4 and annealed CND

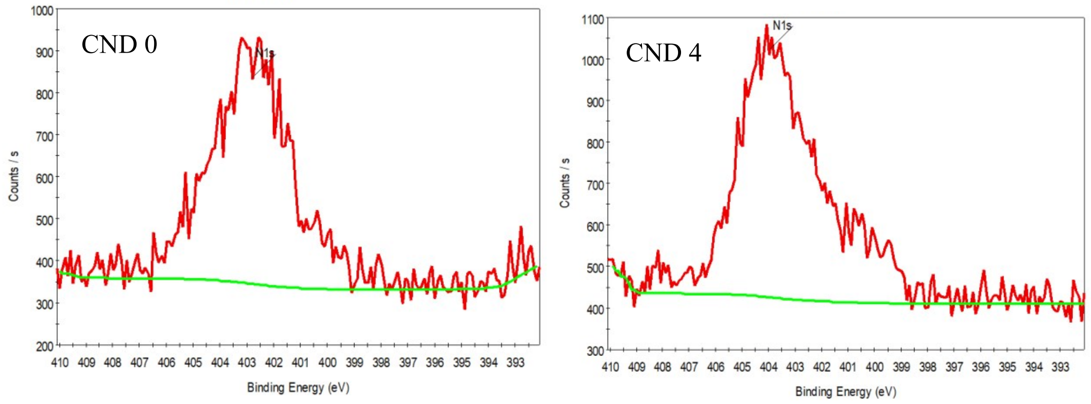

Figure S9. Representative XPS N1s spectra of CND 0 and CND 4. No significant change is obtained in N1s spectra of all samples, indicating similar N state.

NMR of N-carbamoyl acetamide

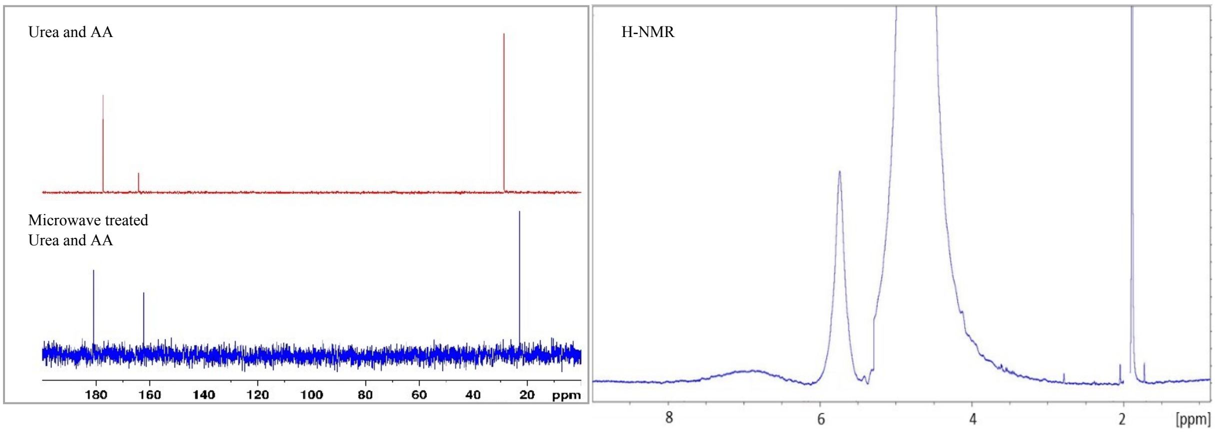

Figure S10 . NMR spectra of N-carbamoyl acetamide formed reaction of urea and acetic acid. *Left* ^{13}C-NMR of untreated (up) and microwave treated (down) urea and AA mixture. *Right* ^{1}H-NMR of microwave treated urea and AA.

To validate the reaction of urea and AA we synthesized the intermediate subjected to the same conditions of the CND synthesis (in absence of citric acid). A comparison of the 13C and 1H NMR spectra of a mixture of urea and acetic acid in H_2O before the reaction and after the reaction are given in the supporting information. Clearly, a new compound is produced after the reaction, based on the difference in the 13C chemical shifts. The methyl group shifted from δ= 22.76 ppm to δ= 20.06 ppm after the reaction, and carbonyl group shifted as well from δ=176.33 to 180.82 ppm after the reaction. The change of the chemical shift of the methyl group is also seen in the 1H-NMR spectrum (from δ=2.02 ppm to δ=1.89 ppm. In addition, the two separate signals originated from hydrogens on two different nitrogen atoms are seen at δ=5.75 ppm and δ=6.93 ppm. These two signals support the formation of the two-nitrogen molecule, N-carbamoyl acetamide.

Optical behavior of CND 0-5 in solutions with different pH.

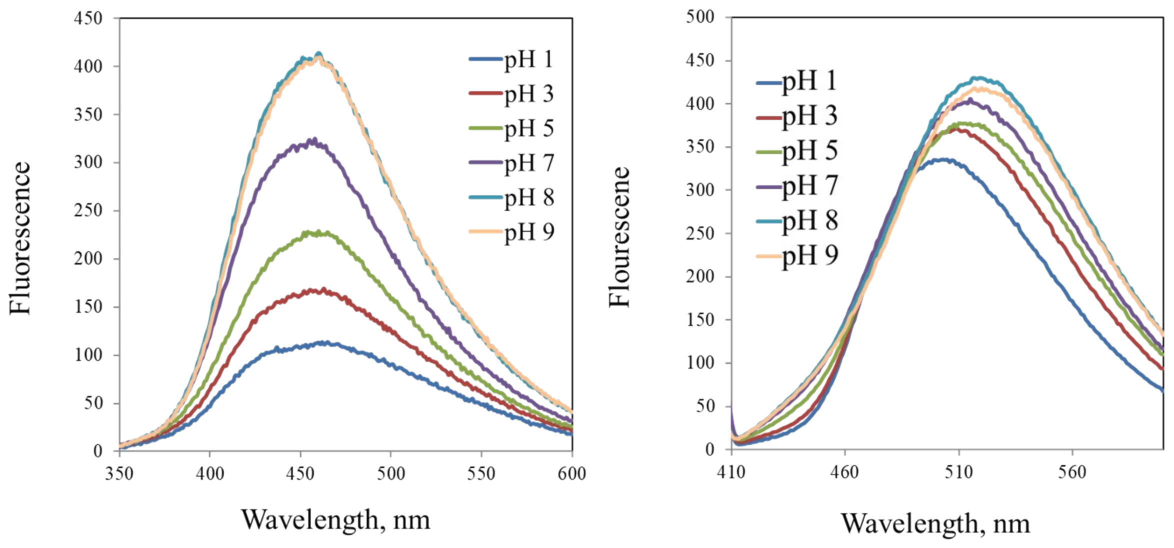

Figure S11.Fluorescence emission (FE) upon excitation wavelengths of 340 nm (left) and 405 nm (right).

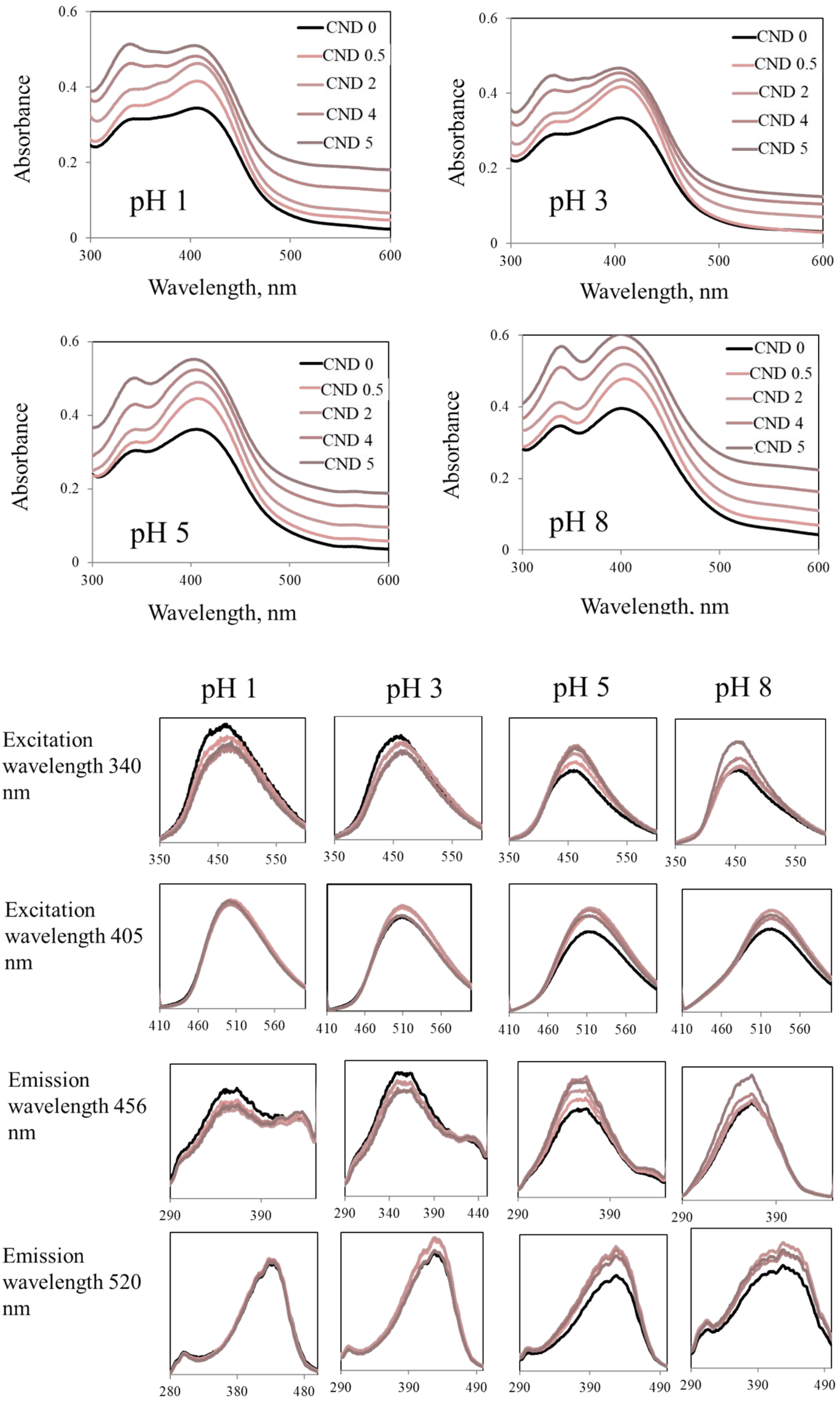

Figure S12. Absorbance, Fluorescence emission (FE) and Photoluminescence excitation (PLE) of CND 0-5 (0- Black, 0.5-5 from light to dark) in different pH aqueous solutions.

Recovery of optical behavior after neutralizing acidity of solvent

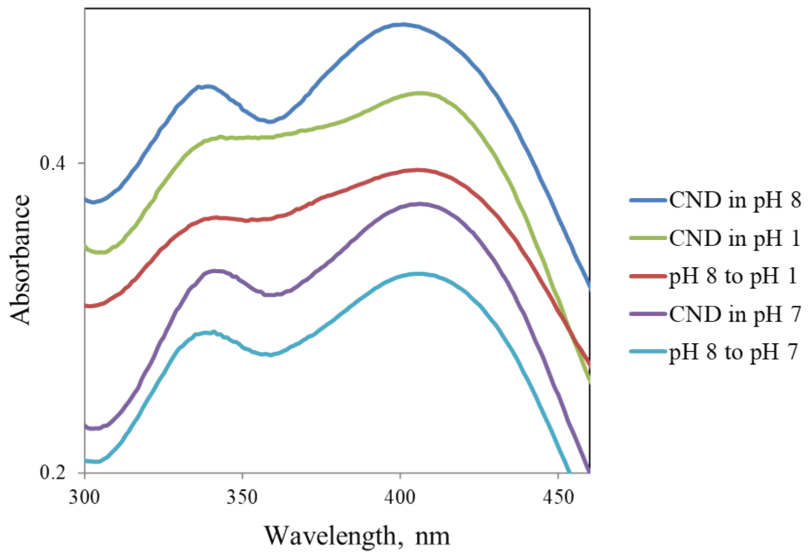

Figure S13. Absorption spectra of CND 0 in basic solution (pH 8) and after neutralizing and acidifying (pH 7 and 1) the solution. The shape of the spectra is not affected by the pH in which the sample was dispersed in previously. It is affected only by the pH of the current solution. This proves no irreversible chemical change has been occurring in the dispersed CNDs even at harsh acidities.

XPS for annealed CND

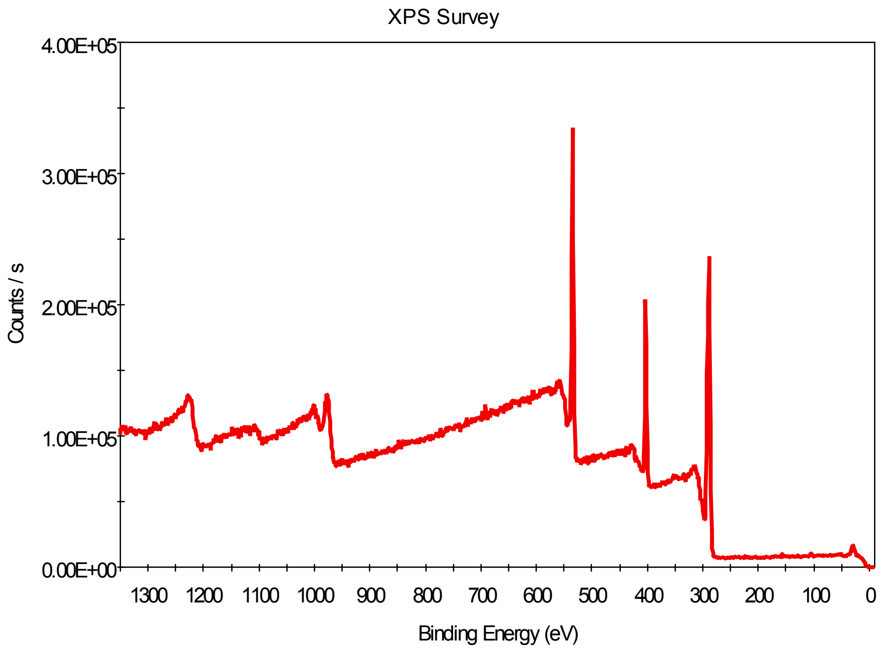

Figure S14. Full XPS spectra of annealed CND.

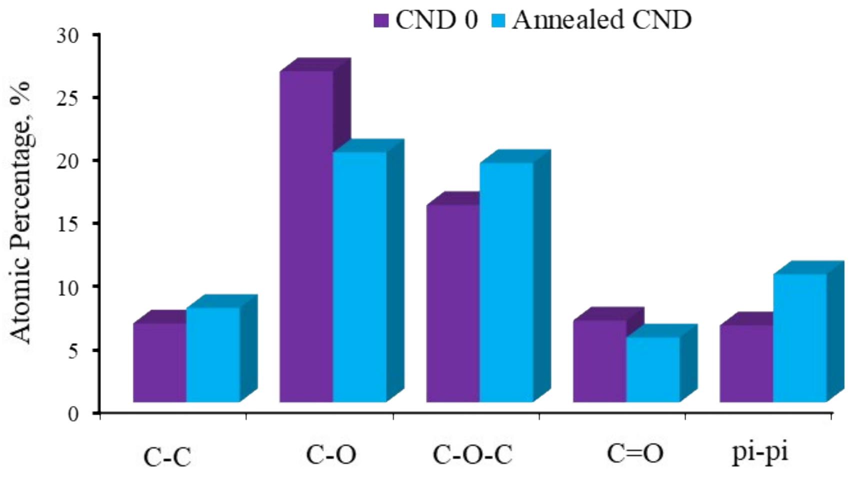

Figure S15.Qualitative splitting of the C1S peak area of CND 0 and annealed CND

Acetic acid assisted HPPT formation mechanism

With acetic acid Without acetic acid

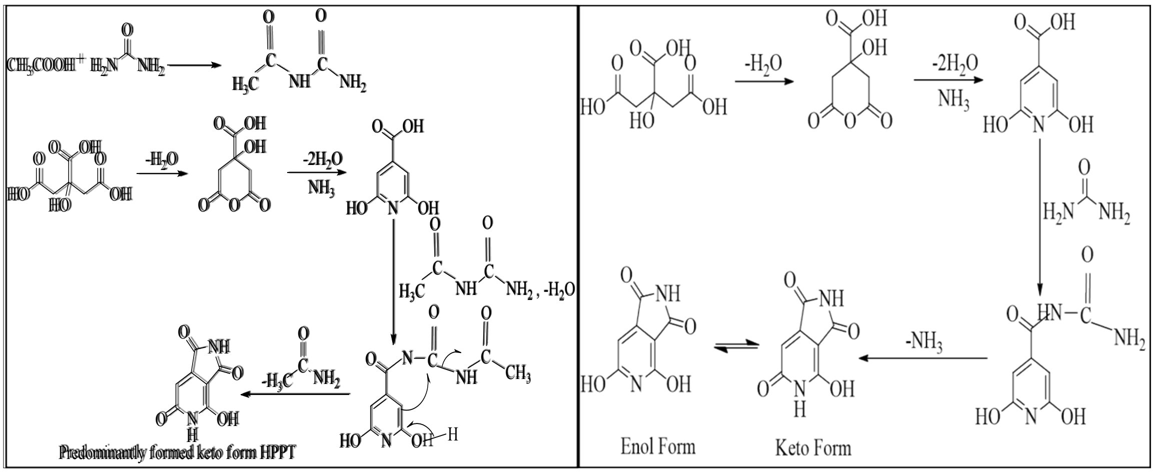

Scheme S2. HPPT formation mechanism in presence (right) and absence (left) of acetic acid.

The mechanisms HPPT formation with and without acetic acid start similarly. Citric acid losses three water molecules (subsequent steps) and substitution of ammonia molecule (dehydrated urea) forms citrazinic acid. At this step, there is a difference between the reactions in presence and absence of acetic acid. In absence of acetic acid, the citrazinic acid reacts with urea. A second cyclization reaction takes place when the nucleophilic carbon 3-position of the pyridine ring attacks the electrophilic carbon on the side chain ($\underline{\mathbf{C}}ONH_2$). With removal of ammonia, the product is HPPT with both the keto and enol forms. This mechanism is described in ref. 39. When acetic acid is added, it reacts, in a separate reaction, with urea to form N-carbamoylacetamide. This molecule then reacts with the citrazinic acid (in a similar way that urea reacts in absence of AA) to form intermolecular amide (See figure). Here the electrophilic carbon on the chain is more positive due to the more electron withdrawing side chain. Additionally, the nucleophilic 3-position carbon is less electronegative. Hence, in order to the nucleophilic attack to occur, an additionally electronegativity should be applied on the nucleophilic carbon. The extra electron density in gained by release of a proton and formation of oxygen double bond to the 2-potision carbon on the 2- pyridone ring. In this case, keto form of HPPT is predominantly formed.
